# Clinical-Pathological Conference Series from the Medical University of Graz: Case No 168: A 28-year-old Syrian refugee with severe abdominal pain and eosinophilia

**DOI:** 10.1007/s00508-018-1395-z

**Published:** 2018-10-15

**Authors:** Elisabeth Fabian, Herbert Auer, Patrizia Kump, Robert Krause, Martin Wagner, Michael Fuchsjäger, Elmar Janek, Horst Olschewski, Guenter J. Krejs

**Affiliations:** 1 Division of Gastroenterology and Hepatology, Department of Internal Medicine III, Medical University of Vienna, Vienna, Austria; 2 Department for Medical Parasitology, Institute of Specific Prophylaxis and Tropical Medicine, Center for Pathophysiology, Infectiology and Immunology, Medical University of Vienna, Vienna, Austria; 3 Division of Gastroenterology and Hepatology, Department of Internal Medicine, Medical University of Graz, Auenbruggerplatz 15, 8036 Graz, Austria; 4 Section of Infectious Diseases and Tropical Medicine, Department of Internal Medicine, Medical University of Graz, Graz, Austria; 5 Division of General Radiology, Department of Radiology, Medical University of Graz, Graz, Austria; 6 Division of Pulmonary Medicine, Department of Internal Medicine, Medical University of Graz, Graz, Austria

**Keywords:** Strongyloides stercoralis, Strongyloidiasis, Hyperinfection syndrome, Abdominal pain

## Presentation of case

### Dr. P. Kump:

**Fig. 1:**
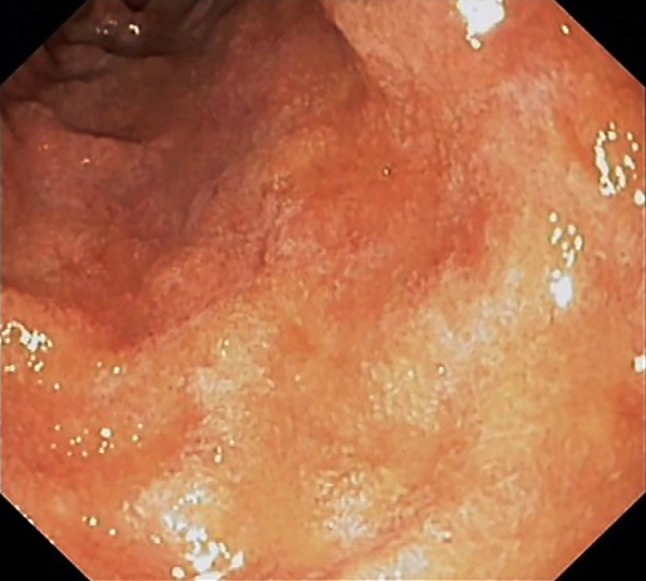
Coarse mucosa with blunted folds in pars I of the duodenum

When the patient, a Syrian refugee, presented at the emergency department of the Medical University of Graz, he had already been in Austria for 2.5 years. A language barrier made it difficult to obtain a complete history. The patient had sought medical attention 3 days earlier at another hospital for epigastric pain of 2 weeks duration that increased after meals. Pantoprazole and sucralfate did not alleviate the epigastric pain. The patient presented at the emergency department of the Medical University of Graz with persistent abdominal pain 1 day prior to admission. Physical examination revealed blood pressure of 125/81 mm Hg, a regular pulse of 60 per min, temperature 35.0 ℃, soft abdominal wall and epigastric tenderness on deep palpation. Except for eosinophils 7% (normal: up to 5%), eosinophils absolute 0.7 × 10^9^/l (normal: up to 0.7 × 10^9^/l) and lactate dehydrogenase (LDH) 287 U/l (normal: 120–240 U/l), laboratory results were unremarkable. The C‑reactive protein (CRP) level was 1.2 mg/l (normal: up to 5.0 mg/l) and liver function tests were normal. Ultrasonography of the liver, the biliary tree, the spleen and the kidneys was unremarkable; the pancreas was not seen. Gastroscopy showed patchy erythema in the antrum, with minimal chronic inactive gastritis on histology. Biopsies from the antrum and corpus were negative for *Helicobacter pylori*. There were coarse folds in the duodenum and on endoscopic magnification the villous structures appeared to be diminished (Fig. 1). Since the patient was uncooperative and increasingly agitated, the procedure had to be terminated before duodenal biopsies could be obtained. Antibodies for serum anti-tissue transglutaminase were negative. The patient was sent home and advised to continue the proton pump inhibitor at double the standard dose and to take scopalamine butylbromide as needed. The next day, however, the patient returned when a banana he had eaten exacerbated the abdominal pain. Bowel sounds were hyperactive, otherwise physical examination was unchanged. Laboratory tests: leukocytes 13.4 × 10^9^/l (normal: 4.4–11.3 × 10^9^/l), eosinophils 9%, eosinophils absolute 1.2 × 10^9^/l, CRP 6.0 mg/l. Since treatment with i.v. esomeprazole, metamizole, scopalamine butylbromide, and fluids did not improve the discomfort, the patient was admitted to hospital. Subsequently, the abdominal pain shifted from the epigastrium to the lower abdomen. On physical examination the consulting gastroenterologist found a positive McBurney sign, a contralateral rebound and significant gas bloating. Computed tomography (CT) of the abdomen showed unremarkable parenchymatous organs, a few air-fluid levels in the colon and enlarged mesenteric lymph nodes (up to 1 cm) in the left upper quadrant thought to be inflammatory in nature. The appendix was unremarkable and there were no signs of diverticulitis. A surgical consultant saw no need for exploratory laparotomy to clarify an acute abdomen.

Stool analysis was negative for *Clostridium difficile* antigen and toxins A and B, *Cryptosporidium *spp. and amoeba. Results in serum were negative or normal for antinuclear antibodies (ANA), nuc180, cytoplasmatic antibodies, extractable nuclear antibodies (ENA) screening, antibodies against double stranded DNA, myeloperoxidase anti-neutrophil cytoplasmatic antibodies (mpo-ANCA), perinuclear ANCA (p-ANCA), proteinase 3 ANCA (pr3-ANCA), cytoplasmatic ANCA (c-ANCA) and immunoglobulin (Ig)A.

During the next 3 days the abdominal pain was accompanied by at least three mushy stools per day and did not improve. Laboratory revealed further increases in leukocytes (16.0 × 10^9^/l) and eosinophils to 12%, 29% and 37% (last absolute eosinophil count: 6.0 × 10^9^/l). The results of a diagnostic test became available.

## Differential diagnosis

### Dr. H. Auer:

The patient is a young refugee from Syria who suffered from abdominal pain that increased in severity over several days. Since the condition did not improve with the medication received, the patient was admitted to hospital, where leukocytes, eosinophils and CRP continued to rise. Stool analysis was negative for common enteric pathogens.

Regarding the patient’s eosinophilia, different underlying causes have to be considered. Generally, elevated eosinophils can be due to their recruitment to a specific site in the body or an increased production of the cells in the bone marrow. Eosinophilia can have many causes, including parasitic and fungal diseases, allergies, adrenal abnormalities, skin disorders, toxins, autoimmune disorders, other endocrine disorders and tumors. Moreover, eosinophilia may be caused by various specific diseases, such as asthma, acute myelogenous leukemia, hypereosinophilic syndrome and inflammatory bowel disease. Parasitic diseases and allergic reactions are, however, among the more common causes of eosinophilia [1].

The clinical details and Syria as the patient’s country of origin could suggest a parasitic infection. Infections with various parasites may be underestimated in countries with high standards of hygiene but worldwide millions of humans are affected. The discussed patient presented with eosinophilia, severe abdominal pain and at least three mushy but not bloody or mucous stools per day. This raises the questions: Which parasitic infection could be responsible for this condition? Which parasites colonize, live and reproduce in the human gastrointestinal tract and thereby cause these symptoms?

Protozoa: Infection with the protozoan parasite *Entamoeba histolytica* should always be considered when a patient presents with abdominal symptoms and bloody or mucous diarrhea. While eosinophilia is not typical for this condition, it is, however, a hallmark for infections with *Giardia lamblia*, which can be detected under the microscope, by antigen detection tests and enzyme immunoassays or by immunofluorescence, with PCR used for further subtyping. Infections with this protozoan parasite, which are acquired orally and mostly via uptake of cysts in contaminated drinking water, are responsible for more than 280 million cases of giardiasis worldwide every year [2]. After transformation to the trophozoite stage, parasites start to colonize the duodenum and jejunum. *Giardia lamblia* attaches to the intestinal epithelium and disrupts epithelial barrier function by altering tight junction composition and increasing apoptosis [3]. Symptoms of acute *Giardia* infection include abdominal pain, diarrhea, bloating and greasy stools that tend to float; indigestion or nausea and vomiting are also frequently reported [4–6]. In chronic giardiasis, symptoms fluctuate and steatorrhea may be due to the formation of a layer of *Giardia* trophozoites by attaching with their large ventral sucking disk above the duodenal mucosa. Besides the described cellular and mechanical effects resulting in increased epithelial permeability, *Giardia lamblia* also causes intestinal abnormalities in the host, such as loss of intestinal brush boarder surface area, villus flattening, and inhibition of disaccharidase activity, while it can also promote enteric bacterial overgrowth [7].

*Cryptosporidium *spp. is the protozoan parasite which caused the most food and waterborne disease outbreaks worldwide during 2004–2010 [8]. *Cryptosporidium parvum, C. hominis *and *C. meleagridis* are the most common species of the genus *Cryptosporidium* in humans, with *C. parvum *and *C.* hominis being responsible for more than 90% of cryptosporidiosis cases [9]. This disease frequently occurs in tropical and subtropical countries [10]. Due to climate changes entailing a higher frequency of heavy rainfalls or floods, the incidence of cryptosporidiosis has also been rising in Europe in recent years with *C. hominis* as the most common pathogen [11]. The leading symptom of cryptosporidiosis is severe watery diarrhea; other frequent symptoms are stomach cramps and pain, nausea, vomiting, dehydration and fever [12]. Infection with *Cryptosporidium *spp. is usually detected by microscopic examination of stool specimens using different preparation techniques, such as acid-fast staining, direct fluorescent antibodies and/or enzyme immunoassays to detect *Cryptosporidium* spp. antigens [10]. *Cyclospora cayetanensis* is a protozoon that is closely related to *Cryptosporidium. *Infection occurs only sporadically in humans and causes only a minimal increase in eosinophils. *Sarcocystis* species are intracellular protozoan parasites with an intermediate-definitive host life cycle based on a prey-predator relationship. Most *Sarcocystis* species infect specific hosts or closely related host species. Humans are definitive hosts for *S. hominis* and *S. suihominis* and can be infected by eating raw beef or pork [13]. Since this disease is usually self-limiting after 3–5 days, most cases go unreported. For muscular sarcocystosis only a limited number of cases in tropical and subtropical environments have been reported [14].* Cystoisospora belli *(formerly known as *Isospora belli*) is a coccidian parasite most commonly found in tropical and subtropical areas. Cystoisosporiasis is caused by ingestion of contaminated food or water and characteristically presents with watery, nonbloody diarrhea. In contrast to other protozoan infections, eosinophilia may be present [15]. *Balantidium coli *is a large ciliated protozoan parasite that can infect humans through the fecal-oral route by contaminated food and water. Balantidiosis is an uncommon human disease mostly restricted to tropical and subtropical regions. Since pigs are an animal reservoir, human infections are also more frequently reported in areas where pigs are raised. Most patients are asymptomatic but in debilitated persons symptoms, such as persistent diarrhea, dysentery and abdominal pain can be severe [16, 17].

Although the discussed protozoan parasites all cause various gastrointestinal symptoms and some of them even eosinophilia, none of these infections is really compatible with the clinical presentation of this patient. Moreover, infection with *Entamoeba histolytica* and *Cryptosporidium *spp. can almost certainly be excluded as a diagnosis due to negative stool analysis; however, there are also various helminths that characteristically cause eosinophilia and could have been responsible for this patient’s condition.

Helminths: Specifically regarding the patient’s Syrian/Middle East origin, an infection with *Schistosoma mansoni* or *S. haematobium* has to be considered. Other species such as *S. japonicum, S. mekongi* and *S. intercalatum *are found in the Far East, Southeast Asia, and West Africa [18]. Schistosomiasis, which is also known as Katayama’s fever, affects at least 230 million people worldwide [19]. In this disease the immune system overreacts to the eggs, cercariae, schistosomulae and adult worms, leading to egg granulomas, cercarial dermatitis, vasculitis and endophlebitis. The clinical presentation of acute schistosomiasis includes fever, headache, abdominal pain, diarrhea, hepatosplenomegaly, myalgia, malaise, fatigue and eosinophilia [18–20]. Since these symptoms can, however, occur weeks after the initial infection and chronic manifestations (nonspecific intermittent rectal bleeding, abdominal pain and diarrhea [19]) were not observed, this disease can be ruled out as the patient had already been in Austria for 2.5 years.

*Fascioloa hepatica* is a liver trematode that has historically been endemic in the Andean countries, the Caribbean, the Caspian region, northern Africa, and western Europe, but it is now spread globally [20]. Acute intrahepatic fascioliasis is caused by the migration of larvae from the intestine to the liver and includes symptoms such as fever, vomiting, abdominal pain, diarrhea, urticaria, and hepatomegaly; leukocytosis and eosinophila are also typical for this disease. In the chronic phase the flukes localize to the bile ducts, thereby causing intermittent biliary obstruction and inflammation; thus, liver function tests are usually abnormal [21, 22]. Migration of the parasite to other organs, such as the brain, muscles and eyes, results in ectopic fascioliasis presenting with nonspecific symptoms [23]. Although fascioliasis is widespread and has become a significant public health problem with over 20 million cases being reported worldwide [24], the symptoms of the discussed patient are not compatible with those typical for this disease and liver function tests were also normal.

*Diphyllobothrium latum, *also called broad tapeworm or fish tapeworm, has long been known as a human intestinal parasite. The infection is transferred to humans by consumption of contaminated raw, smoked, pickled or poorly cooked fish, which are fundamental reservoirs of *Diphyllobothrium* because plerocercoids may survive in their body for months or years [25]. Most commonly, intermediate hosts of *Diphyllobothrium latum *are perch, pike and burbot in Europe and pikeperch or walleye in North America [26]. Salmonoids and whitefish have also been reported to transfer the infection to humans [25, 27]. Growing up to 2‑15 m (reported maximum length 25 m) in length as adults, *Diphyllobotrium* tapeworms are among the largest human parasites [28]. The growth rate of the worm is about 22 cm/day or almost 1 cm/h and adult worms may survive decades in the human gastrointestinal tract [29]. Infection is often asymptomatic and only one out of five patients experiences manifestations such as diarrhea, abdominal pain or discomfort, fatigue, partial obstruction or megaloblastic anemia [25]. Eosinophilia is occasionally observed in diphyllobothriasis [30].

Soil-transmitted helminths, such as *Ascaris lumbricoides, Trichuris trichiura*, and hookworms (*Necator americanus* and *Ancylostoma duodenale*) cumulatively cause the most widespread neglected tropical disease with nearly 1.5 billion people infected with at least 1 nematode in over 100 endemic countries [31, 32]. Due to their widespread occurrence, infection with these helminths should also be considered and ruled out in this case.

*Ascaris lumbricoides *is a parasite with an adult length of about 30–40 cm and a lifespan of 6–18 months; its habitat is the human jejunum [33]. *A. lumbricoides* infects more than 800 million people worldwide, especially in the Indian subcontinent, China, Africa and Latin America. Ascariasis is uncommon in Europe and in the USA, where most documented cases involve immigrants from developing countries [34, 35]. The majority of patients with ascaris infections are asymptomatic. Clinical manifestations are restricted to a small percentage of those with a heavy worm burden (e.g. intestinal obstruction causing bowel infarction and gangrene) or when the worm migrates to other organs, causing such conditions as Ascaris pneumonia, peritoneal, gastric, hepatobiliary or pancreatic ascariasis. It is estimated that about 20,000 deaths per year are due to such a severe clinical disease course [33]. In the discussed patient ascariasis is, however, unlikely because of the clinical presentation, the abdominal imaging studies with no worms in the intestinal lumen and the negative stool analysis. Furthermore, a hookworm infection that would cause an increase in eosinophils can also be ruled out in this case, because it would primarily present with anemia and nonspecific gastrointestinal symptoms but not as acute abdomen. *Trichuris trichiura*, also known as human whipworm, is the third most common roundworm in humans. Worldwide about 800 million people suffer from trichuriasis. Most frequently the infection is asymptomatic; in some cases it may cause nonspecific gastrointestinal symptoms such as abdominal pain and diarrhea [36]. Infection with* Trichuris trichiura* was detected in the Tyrolean Iceman who lived about 5300 years ago [37] but is unlikely to be responsible for this patient’s symptoms.

Finally, infection with *Strongyloides stercoralis*, one of the most common and globally distributed human pathogens of clinical importance, should also be considered. Currently, it is estimated that about 30–100 million people are infected worldwide [38]. Although the global prevalence has increased during the past 5 years, strongyloidiasis is underestimated in many tropical and subtropical countries. Poor personal hygiene, insufficient drinking water supply, unsatisfactory sanitation, and lack of information about the disease are important factors contributing to an increased prevalence in endemic countries [39]. Infection of the upper gastrointestinal tract with *Strongyloides stercoralis* is often asymptomatic or causes only minimal gastrointestinal symptoms, although this nematode is able to persist and replicate within the host for decades. Strongyloidiasis may, however, become life-threatening due to dissemination and hyperinfection in patients undergoing immunosuppressive or corticosteroid therapy, organ transplant recipients, and patients with hematological malignancies or other debilitating diseases [39]. Some cases of disseminated disease with bloody pericardial effusion [40] or hyperinfection syndrome have also been described in immunocompetent patients [41]. Hyperinfection can involve any organs but typically affects the gastrointestinal tract and the lungs. In this state worms can be found in extraintestinal regions and highly infective larvae are detectable in body secretions and excreta [42]. Mortality is up to 87% with this syndrome [43]. Thus, awareness for parasite screening prior to empirical corticosteroid therapy in immunosuppressed high-risk patients is of utmost importance. This is also confirmed by my personal experience: just recently I was consulted as a parasitologist because of hyperinfection with *Strongyloides stercoralis* in an immunosuppressed organ transplant recipient from Turkey. When organ rejection was imminent he received corticosteroids and the condition markedly deteriorated. Infection with *Strongyloides stercoralis* was finally diagnosed by detection of larvae on histology of the duodenal mucosa. Strongyloides was also found in his bronchial lavage. With albendazole the patient recovered fully.

Further parasites that might have to be considered in the discussed patient include *Trichinella *spp. and *Anisakis simplex*. Both parasites can be transmitted to humans through consumption of infected raw meat or raw or pickled fish. Although the clinical manifestations include abdominal pain and eosinophilia, as observed in this patient, an infection seems unlikely because he had not eaten raw meat or fish. Infections with *Toxocara canis, Toxocara cati* or* Ascaris suum* are also frequently observed in humans. These infections are associated with increased eosinophils [44, 45] and visceral larva migrans (larva migrans visceralis syndrome sensu lato), a condition that was first reported in 1952 [46] and is caused by migration of larvae of nematodes that can infect but not mature in humans (dead-end host). After penetration of the intestinal wall larvae can spread in the body via the bloodstream and subsequently cause inflammation and damage to various organs. Since CT imaging did not suggest organ damage by migrated larvae and since gastrointestinal symptoms as described in the protocol are only rarely observed in these infections, I would rule them out as diagnosis here.

To establish the final diagnosis, further stool and/or antigen analysis should be done. Considering the clinical manifestations, leukocytosis and eosinophila, and also the fact that the patient had already been in Austria for more than 2 years (i.e. the symptoms could be due to a long persisting parasite) it can be suggested that additional analyses would most likely detect infection with *Strongyloides stercoralis*.

## Dr. H. Auer’s diagnosis

Strongyloidiasis

## Discussion of case

### Dr. R. Krause:

In this case further microscopic stool analysis detected larvae but no eggs of *Strongyloides stercoralis*. Ivermectin therapy was initiated orally at 200 µg/kg/day for 2 days; this usually results in high degree of parasite eradication [47]. Ivermectin is the drug of choice in strongyloidiasis because it has fewer unfavorable side effects and a better rate of larval clearance from stool than thiabendazole and albendazole [48, 49]. The drug is available in oral or parenteral form but the Food and Drug Administration has approved only the oral formulation for human use [50]. According to the US Center for Disease Control and Prevention, veterinary subcutaneous ivermectin may be a therapeutic option when oral and/or rectal administration of the drug is not possible. Although the current literature on humans is limited there are some reports of successful subcutaneous ivermectin treatment for strongyloidiasis in immunocompromized patients with disseminated disease [51] and hyperinfection [52].

### Dr. H. Auer:

*Strongyloides stercoralis* is an intestinal parasite of vertebrates and exhibits a complex and unique developmental phase with a free-living and a parasitic part of its life cycle completed in the same host [39]. In the free-living phase male and female adults develop in the soil. In the parasitic phase noninfective larvae enter the host by penetrating the skin or by the fecal-oral route to moult to infective filariform larvae. At their portal of entry, larvae often cause a petechial hemorrhagic rash, pruritus, and local edema. After entry the larvae migrate to the pulmonary circulation through lymphatics and venules. There they move to the alveolar spaces and cause inflammation associated with eosinophilia; they then crawl up the respiratory tract and are swallowed, thereby reaching the intestine. Other options for larvae to enter the gastrointestinal tract are the fecal-oral route or autoinfection. Once the small bowel is reached larvae mature into adult females and produce eggs via parthenogenesis. The eggs hatch in the intestine into noninfective rhabditiform larvae that are excreted and continue the free-living life cycle. Premature transformation of noninfective to infective larvae allows penetration of the mucosa of the small bowel or colon, or the larvae penetrate the perianal skin, thus starting a new developmental phase within the host, i.e. causing autoinfection. This specific ability of *Strongyloides stercoralis* for repeated migratory cycles within the primary host is responsible for the increase in the worm burden without exogenous reinfection and the decade-long persistence of the infection in untreated individuals.

### Dr. G.J. Krejs:

**Fig. 2:**
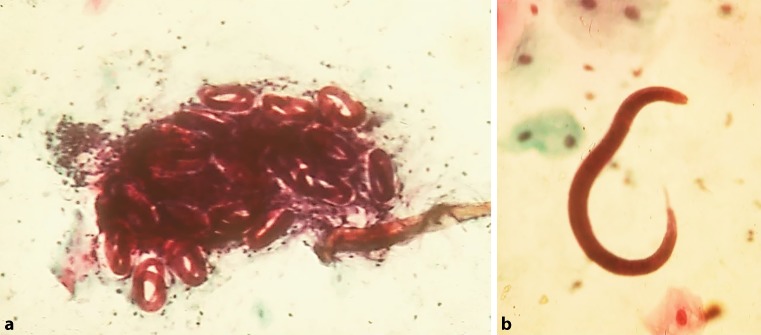
Giemsa stain of brush cytology of duodenal mucosa in a patient with chronic asthma and arthritis who received 80 mg of prednisone daily, showing a cluster of strongyloides larvae (✕ 50) (**a**) and a single larva on higher magnification (✕ 125) (**b**). (Personal observation of senior author together with Dr. Stuart Frank, Dallas, Texas 1977)

In Europe where hygiene standards are generally high, parasite infection is usually found in patients who migrated from or travelled to endemic countries; however, some cases of autochthonous infections have also been reported [53]. In Austria, strongyloidiasis is rare and in over 30 years of patient care in this department I can remember just one case of autochthonous strongyloidiasis. During my time at Southwestern Medical School in Dallas, Texas, I diagnosed several cases of strongyloidiasis. Characteristic findings with upper gastrointestinal endoscopy (Fig. 1) include gastric mucosal erythema and areas of atrophy. The duodenal mucosa presents with a coarse surface, enlarged and rigid villous structures, diffuse erythema, edema and friability. Duodenal biopsy may reveal larval infection. Fig. 2 (personal observation) shows brush cytology from the duodenal mucosa. In the discussed patient the endoscopic finding of an altered mucosal surface and altered villous appearance in the duodenum could have suggested strongyloidiasis, but since the patient’s noncompliance precluded a biopsy, the infection could not be confirmed histologically. Years ago I was the attending physician of a 32-year-old man working as a heavy machine operator for an oil company. He had been in Nigeria to lay pipelines in a swampy jungle area and back in the United States presented with fever, truncal rash, nausea, vomiting, and diarrhea with up to 12 bowel movements per day. In fact, epigastric pain and watery diarrhea are the most common gastrointestinal manifestations of strongyloidiasis, which was first described in 1876 in French soldiers returning from the old Indochina region and therefore was called Cochin-China diarrhea [54]. Treatment with opiates did not improve my patient’s condition. The patient became dizzy and complained of chest pain 6 weeks after the onset of diarrhea, which was due to an electrolyte imbalance/hypokalemia (serum potassium: 2.8 mEq/l) and consequently led to cardiac arrest. Finally, the patient was diagnosed and treated for strongyloidiasis (18% eosinophils). Perfusion studies revealed increased secretion instead of absorption of water and electrolytes in the jejunum, which had been responsible for the persisting diarrhea and subsequently hypokalemia and cardiac arrest [55].

When a patient at risk presents with gastrointestinal symptoms and upper gastrointestinal endoscopy reveals alterations of the gastric and duodenal mucosa, gastroenterologists should think of strongyloidiasis. The importance of diagnosing this disease is further emphasized by scientific presentations at recent annual congresses, such as the 2017 World Congress of Gastroenterology in Orlando, Florida, and the United European Gastroenterology Week in Barcelona.

### Dr. P. Kump:

In this case the gastroenterologist was primarily consulted because of severe abdominal pain. The patient reported mushy stools but not diarrhea, so abdominal pain was the leading symptom. Physical examination first suggested appendicitis but this could be ruled out when the abdominal CT was unremarkable and laboratory results showed an increased eosinophil count. Abdominal pain and eosinophilia in a patient who had migrated from a country with a high prevalence of parasitic diseases strongly hinted to a parasitic infection. To confirm this suspicion stool analyses were performed and larvae of *Strongyloides stercoralis* were finally detected. After therapy with ivermectin the abdominal pain resolved promptly and eosinophilia disappeared. Stool examination for parasites was negative 3 and 7 days following ivermectin.

### Dr. M. Fuchsjäger:

As already mentioned abdominal CT was unremarkable. Single air-fluid levels in the transverse colon and enlarged mesenteric lymph nodes up to 1 cm in size, are signs for a degree of irritation and mild inflammation of the gut. As described by Dr. Krejs, infection with *Strongyloides stercoralis* can shift gastrointestinal water absorption to secretion. This pathophysiology may have resulted in the radiological finding of air-fluid levels in the gut.

### Dr. D. Schiller (by e‑mail):

Considering the clinical presentation (with the possibility of a long period between infection and onset of symptoms), the upper gastrointestinal endoscopy findings and the increased eosinophil count in a patient from a region where *Strongyloides stercoralis* is endemic, my diagnosis is strongyloidiasis.

### Dr. G.J. Krejs:

Among 168 clinical-pathological cases in the last 31 years in this institution, 6 cases with infection by helminths have been discussed. These include one case of strongyloidiasis, one of fascioliasis, two cases of echinococcosis, one case of infection with *Ancylostoma duodenalis*, and one case with *Clonorchis sinensis*.

An interesting question that remains is whether the administration of proton pump inhibitors exacerbated the patient’s disease and whether the increasing eosinophil count might have indicated that he was on the way to a hyperinfection state.

### Dr. D. Schiller:

Besides organ recipients, today many other patients are treated with immunosuppressive drugs for a variety of diseases. Since dissemination and hyperinfection syndrome caused by infection with *Strongyloides stercoralis* have a very high mortality, routine parasite screening should probably be done prior to immunosuppressive therapy in high-risk patients.

### Dr. H. Auer:

Routine parasite screening would be useful in high-risk patients treated with immunosuppressives. Currently, not only microscopic stool analysis but also specific blood tests are available for detecting several parasites including *Strongyloides stercoralis*. So far as I know, however, such screening is rarely done and disseminated strongyloidiasis and hyperinfection are frequently misdiagnosed as gram-negative septicemia, acute respiratory distress syndrome, or multiorgan failure. Finally, this instructive case clearly showed that parasitic infections can present with a wide range of clinical manifestations that are not necessarily given in a textbook. This means that whenever an infection is suspected a comprehensive parasite screening should be ordered.

## Final diagnosis

Strongyloidiasis
